# Regional differences in fetal fat accretion in small-for-gestational-age fetuses assessed by quantitative magnetic resonance imaging

**DOI:** 10.1007/s00247-026-06702-2

**Published:** 2026-07-01

**Authors:** Bar Neeman, Levi Elhadad, Tamir Graziani, Jayan Khawaja, Ayala Zilberman, Sharon Vanetik, Yair Wexler, Jacky Herzlich, Karina Krajden Haratz, Liat Ben Sira, Liran Hiersch, Leo Joskowicz, Dafna Ben Bashat, Aviad Rabinowich

**Affiliations:** 1 Department of Radiology, Tel Aviv Sourasky University Medical Center (Ichilov), Weizman 6, Tel Aviv, 6423906 Israel; 2 Gray Faculty of Medical and Health Sciences, Tel Aviv University, Tel Aviv, Israel; 3 Department of Internal Medicine, Elmhurst Hospital Center, Icahn School of Medicine at Mount Sinai, New York, United States; 4 Department of Obstetrics and Gynecology, Lis Maternity and Women’s Hospital, Tel Aviv Sourasky University Medical Center (Ichilov), Tel Aviv, Israel; 5 Department of Pediatrics, Dana Dwek Children’s Hospital, Tel Aviv Sourasky University Medical Center (Ichilov), Tel Aviv, Israel; 6 School of Neurobiology, Biochemistry and Biophysics, The George S. Wise Faculty of Life Sciences, Tel Aviv University, Tel Aviv, Israel; 7 Neonatal Intensive Care Unit, Dana Dwek Children’s Hospital, Tel Aviv Sourasky University Medical Center (Ichilov), Tel Aviv, Israel; 8 Division of Ultrasound in Obstetrics and Gynecology, Lis Maternity and Women’s Hospital, Tel Aviv Sourasky University Medical Center (Ichilov), Tel Aviv, Israel; 9 Department of Maternal-Fetal Medicine, Lis Maternity and Women’s Hospital, Tel Aviv Sourasky University Medical Center (Ichilov), Tel Aviv, Israel; 10 School of Computer Science and Engineering, Hebrew University of Jerusalem, Jerusalem, Israel; 11 Sagol Brain Institute, Tel Aviv Sourasky University Medical Center (Ichilov), Tel Aviv, Israel; 12 Gray Faculty of Medical and Health Sciences and Sagol School of Neuroscience, Tel Aviv University, Tel Aviv, Israel

**Keywords:** Adipose tissue, Body composition, Fetal growth retardation, Fetus, Magnetic resonance imaging

## Abstract

**Background:**

Fetal fat accretion follows a spatiotemporal pattern. Fetuses who are small-for-gestational-age (SGA) demonstrate reduced fat accumulation, but whether specific body regions are disproportionately affected remains unclear.

**Objective:**

To characterize regional fat differences between SGA and appropriate-for-gestational-age (AGA), assess the modifying effects of SGA-onset timing and cerebroplacental ratio (CPR), and evaluate associations with neonatal morbidity.

**Materials and methods:**

SGA pregnancies were prospectively recruited, and AGA controls retrospectively identified. SGA was defined as estimated fetal weight <10th centile and classified as early-onset (< 32 weeks) or late-onset (≥ 32 weeks), with CPR categorized as normal (≥ 5th centile) or abnormal (< 5th centile). Fetal Magnetic resonance imaging was performed at 3-T using T1-weighted two-point Dixon. Subcutaneous fat was segmented and subdivided into cheeks, trunk, upper and lower limbs. Fat signal fraction and fat mass were computed, with regional fat mass adjusted to global fat mass. Linear mixed models compared groups, and univariate logistic regression assessed associations with adverse outcomes.

**Results:**

Sixty-four participants (35 SGA, 29 AGA) were included. Fat signal fraction was significantly lower in SGA across all regions (*P*<0.001). Upper limb adjusted fat mass was reduced in SGA (*P*=0.043), while other regions showed no differences (*P*≥0.08). Fat signal fraction and adjusted fat mass did not differ by SGA onset or CPR status. Lower fat signal fraction in all regions was associated with higher morbidity rates, whereas adjusted fat mass was not.

**Conclusion:**

SGA fetuses exhibit globally reduced lipid content with a disproportionate upper limb fat mass deficit, suggesting selective vulnerability of peripheral fat depots.

**Graphical Abstract:**

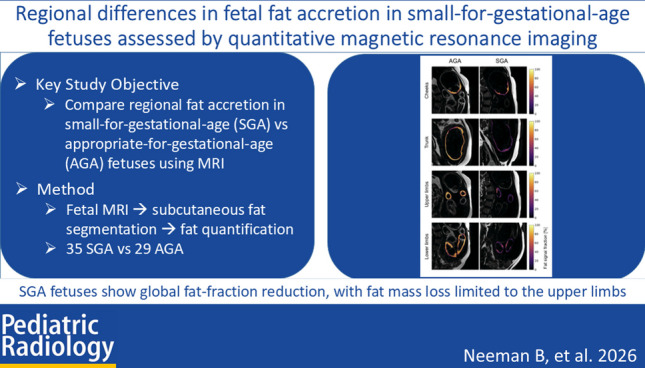

**Supplementary Information:**

The online version contains supplementary material available at 10.1007/s00247-026-06702-2.

## Background

Small-for-gestational-age (SGA) refers to fetuses with an estimated fetal weight below the 10th centile for gestational age and is associated with increased risks of perinatal morbidity and long-term metabolic and cardiovascular disease [1, 2]. SGA fetuses are a heterogeneous population with variable underlying mechanisms and clinical risk, and not all are affected by pathologic growth restriction [3, 4]. Magnetic resonance imaging (MRI)-based studies have shown reduced lipid deposition including subcutaneous fat volume, and fat fraction in fetuses with growth impairment compared with appropriate-for-gestational-age (AGA) fetuses, and a leaner fetal habitus has been associated with adverse perinatal outcomes [3, 5, 6]. These findings support the relevance of fetal body composition assessment in pregnancies complicated by fetal smallness.

Normal fetal fat deposition accelerates during the third trimester. Notably, from the 30th post-conception week, fat accumulation exceeds that of non-fat components [7, 8]; by term, fat constitutes about 16% of human neonate body weight, exceeding any other mammal [9]. The adipose tissue is critical for the neonate’s well-being and supplies the baby with thermal insulation and energy reserves to adapt to extra-uterine life [9]. Typically, subcutaneous fat accretion occurs in a temporal, non-homogenous order. First, fat becomes evident in the cheeks and nuchal region around 26 weeks, followed by the buttocks, thighs and trunk [10]. This pattern probably reflects anatomic priorities for insulation and regional differences in adipose tissue development, as evident on MRI [10].

MRI, particularly fat–water separation techniques like the Dixon method, enables quantitative in utero assessment of fetal adipose tissue [8, 11, 12]. Prior studies have demonstrated that fat–water MRI can be used to detect the typical increase in fat accretion throughout the third trimester, sexual dimorphism in body composition, and significant differences in adipose tissue deposition between fetal growth restriction, SGA and AGA fetuses [3, 7, 8, 10]. Primarily, the fetal fat signal fraction and estimated lipid content are significantly lower among growth restricted fetuses compared to AGA [3]. Thus, MRI constitutes a non-invasive tool to help characterize fetal nutritional status.

A previous work by Giza et al. demonstrated that all fetal subcutaneous compartments increasingly accrete fat throughout the third trimester; however, subregions differ in their lipid fraction, as manifested by the subcutaneous fat proton density fat fraction (PDFF) [8]. The cheeks have the highest fat fraction of 37% around the 30th week of gestation and the upper arms exhibit the highest accretion rate with a 3.5% increase in PDFF per week [8].

However, it remains unknown whether placental-insufficiency related undernutrition preferentially affects specific body regions, and whether disproportionate involvement of particular regions is prognostic of outcome. Moreover, because adipose tissue accretion occurs predominantly in late gestation, the timing of SGA diagnosis may influence patterns of fat distribution. In this context, early-onset SGA, defined as diagnosis before 32 weeks’ gestation, may alter regional fat deposition differently than late-onset SGA. A similar rationale applies to brain sparing, in which the fetus redistributes blood flow to the brain at the expense of peripheral tissues. This process is typically reflected by an abnormal cerebroplacental ratio (CPR) on Doppler ultrasound. Accordingly, fetuses with brain sparing may be expected to exhibit greater impairment of adipose tissue accretion, with potentially disproportionate involvement of specific body regions.

 The objective of this study is to quantify the differences in fat accretion in different subcutaneous compartments between SGA and AGA, between early and late SGA and between SGA fetuses with normal or abnormal Doppler studies. We hypothesized that SGA fetuses show a significant reduction in fat deposition that may be related to specific regions, specifically those with higher fat content or accelerated growth.

## Materials and methods

The Institutional Review Board of Tel Aviv Sourasky University Medical Center approved this study. Participants with SGA and growth restriction-complicated pregnancies were prospectively recruited, and written informed consent was obtained. Participants with AGA pregnancies were retrospectively identified, and informed consent was waived by the institutional committee. Some of the participants included in this study have been previously included in other publications [3, 5, 7, 13]. This study is an extended retrospective analysis focused on additional imaging features and body parts.

### Study population

Participants with SGA and fetal growth restriction-complicated pregnancies were recruited from the Maternal–Fetal Ward at our institution, for simplicity hereafter referred to as SGA. Participants with AGA pregnancies were identified from outpatient referrals to the MRI service at our institution. Table 1 lists the indications for MRI referrals for participants with AGA pregnancies. Participant recruitment was between 2021 and 2024. The inclusion criteria included participants older than 18 years with singleton pregnancies from 28 post-conception weeks. Individuals with fetal chromosomal abnormalities, fetal cytomegalovirus infection, structural anomalies detected on MRI, and body regions with poor image quality were excluded. In addition, body regions with poor image quality were excluded from the corresponding regional analysis. Importantly, poor image quality in one anatomical region did not necessarily lead to exclusion of the entire case. If the remaining images were adequate, the case was retained for analyses of other body regions and for whole-body subcutaneous adipose tissue assessment.

**Table 1:** Indications for magnetic resonance imaging among participants with appropriate for gestational age pregnancies (*n*=29)

Indication	*n*, %
Family history of abnormal pregnancy/born child	6 (21%)
Maternal cytomegalovirus (CMV) seroconversion	4 (13.8%)
Ventriculomegaly or ventricular asymmetry according to ultrasound	4 (13.8%)
Dysmorphic or short corpus callosum according to ultrasound	3 (10.3%)
Maternal toxoplasma infection	2 (6.9%)
Microcephaly according to ultrasound	2 (6.9%)
Other	8 (27.6%)

Ultrasound was performed for all individuals within seven days from the MRI scan. The sonographic estimated fetal weight was computed based on the Hadlock 4 formula [14]. The estimated fetal weight and abdominal circumference were converted to centiles according to Hadlock nomograms [15]. Doppler studies of maternal and fetal vasculature were in accordance with the International Society of Ultrasound in Obstetrics and Gynecology guidelines [16]. SGA was defined as sonographic estimated fetal weight or abdominal circumference below the 10th centile according to [17]. AGA was defined as estimated fetal weight and abdominal circumference between the 10th and 90th centiles. Pregnancies were dated based on first-trimester crown-rump length. To ensure no structural MRI abnormalities, a fetal radiologist (LBS, over 25 years of experienc in fetal imaging) reviewed all cases.

SGA cases were further categorized as early- (diagnosis before the 32nd week of gestation) and late-onset disease (diagnosis onwards of the 32nd week of gestation). Furthermore, cases were categorized according to Doppler studies as normal (CPR ≥ 5th centile) and abnormal (CPR < 5th centile).

Newborns were followed up until hospital discharge, and outcomes were recorded. Neonatal morbidity was defined as the presence of at least one adverse outcome, constituting a composite adverse neonatal outcome. These included: hypoglycemia (plasma glucose <45 mg/dL within the first 24 h after birth), hypothermia (axillary temperature < 36.5 °C/97.7 °F), intraventricular hemorrhage (any grade, per neonatal brain ultrasound), respiratory distress syndrome (based on clinical and imaging findings), necrotizing enterocolitis (any stage, based on clinical and imaging findings), requirement for respiratory support (any modality), > 7 days to achieve full enteral nutrition, or ≥ 10 days of hospitalization in the neonatal intensive care unit (NICU). Due to institutional policy of admitting infants born before 35 post-conception weeks, only hospitalization days beyond the 35th week were counted.

### Fetal magnetic resonance imaging protocol

MRI was performed using 3 Tesla (T) MRI scanners (Prisma, Vida; Siemens Healthineers, Erlangen, Germany) using an 18-phase array abdominal coil and a spine coil embedded in the scanner bed. Participants were positioned supine. The imaging protocol included dedicated fetal brain imaging followed by T_1_-weighted two-point Dixon (T_1_W Dixon) sequence covering the entire uterus, with the following parameters: TR = 4–4.24 ms, TE1 = 1.34 ms, TE2 = 2.57 ms, flip angle = 9 degrees, FOV = 400×400 mm, slice thickness = 2 mm, and in-plane resolution = 1.25–1.4×1.25–1.4 mm, scan time = 18 s, with a single breath hold. Fat and water-only images were automatically generated in real time using the scanner software.

### Image analysis

**Fig. 1:**
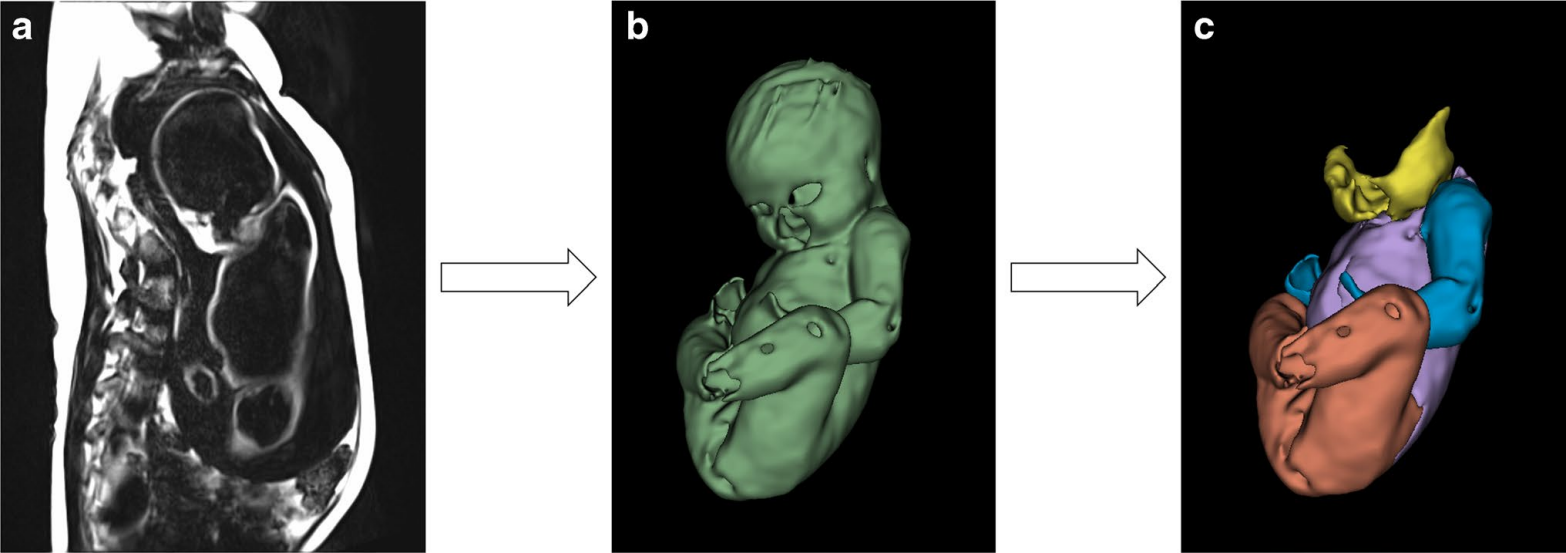
Segmentation of fetal subregional subcutaneous adipose tissue. **a** T_1_-weighted two-point Dixon fat-only image of the entire fetus. **b** 3-dimensional surface rendering of an automatic fetal subcutaneous adipose tissue segmentation. **c** Manual division to fat tissue to subregions: cheeks (*yellow*), trunk (*purple*), upper limbs (*blue*) and lower limbs (*orange*)

The fetal subcutaneous fat tissue was segmented using a previously described in-house automatic deep-learning software [18]. The network architecture, training procedure, and validation performance of this algorithm were previously reported in detail [18]. When needed, manual correction of the segmentations was performed by one of four radiologists (AR, JK, TG, and BN,with 6, 3, 3, and 3 years of experience in image segmentations, respectively) using ITK-SNAP Version 3.8 (https://www.itksnap.org/pmwiki/pmwiki.php) [19]. The total fetal subcutaneous fat was further manually divided to four body regions as follows (Fig. 1): cheeks (extending from the chin to the ears), trunk (including the chest and abdomen, from the base of the neck superiorly to the level where the thighs meet the pelvis inferiorly, and laterally to the shoulders), upper limbs (including both upper extremities from the shoulders to the wrists) and lower limbs (including both lower extremities from the level where the thighs meet the pelvis to the ankles). Subdivision was done using 3D Slicer software Version 5.2 (https://www.slicer.org/) [20] by a radiologist (BN) and a medical student (LE, following dedicated training). Segmentations were then reviewed by an additional radiologist (AR). All reviewers were blinded to the fetal gestational age (GA), sex, AGA/SGA status, ultrasound and MRI findings and referral indication.

### Body composition analysis

Fat and water signal intensities were used to estimate the adipose tissue lipid fraction (in %) according to the following equation:

1 $$fat\ signal\ fraction = \frac{{Fat}_{SI}}{{Fat}_{SI}+{Water}_{SI}}$$

where SI denotes the tissue signal intensity. When referred to as a single measure, fat signal fraction is provided in % (multiplied by 100).

Fat mass (in grams) was calculated using the following equation:

2 $$fat\ mass = {Adipose\ Tissue}_{vol}\times fat\ signal\ fraction\times 0.987$$

where Adipose Tissue_vol_ refers to the subcutaneous adipose tissue volume (in cm^3^). A density factor of 0.987 g/ml was applied based on a previous study [21].

Fat mass was computed for the entire fetal subcutaneous adipose tissue and separately for each region (cheeks, trunk, upper and lower limbs). To account for inherent differences in fetal size between the AGA and SGA groups, regional fat mass values were normalized to total fetal subcutaneous fat mass, expressed as adjusted fat mass:

3 $$adjusted\ fat\ mass=\frac{{fat\ mass}_{region}}{{fat\ mass}_{total}}$$

### Statistical analysis

Statistical analyses were conducted in R and SPSS Statistics version 29.0 (IBM Corp., Armonk, NY). Both adjusted fat mass and fat signal fraction were compared between AGA and SGA groups, and within SGA subgroups defined by timing of diagnosis (early vs. late) and CPR status (normal vs. abnormal), using linear mixed models while accounting for fetal sex and gestational age as covariates, with participant as a random effect. When overall differences were significant, post hoc tests compared the cheeks, trunk, upper and lower limbs. Associations between adjusted fat mass and fat signal fraction and adverse neonatal outcomes were assessed with univariate logistic regression. All p-values presented in this study were grouped and corrected for multiple comparisons using the Benjamini–Hochberg procedure, controlling false discovery rate at the 0.05 level.

## Results

Sixty-four participants with singleton pregnancies were included in the study, comprising 29 (45.3%) with AGA pregnancies and 35 (54.6%) with SGA pregnancies. The average GA at the time of MRI was 34 ± 1.8 weeks among participants with AGA pregnancies and 33.9 ± 1.9 weeks among participants with SGA complicated pregnancies. CPR data were unavailable for one SGA case; of the 34 pregnancies with known CPR data seven (20.6%) had an abnormal CPR. Twenty (57.1%) SGA cases had an early-onset disease. Table 2 lists the participant demographic data. The median postnatal infant follow-up time was 4 days (interquartile range [IQR], 3–10, range 2–25 days), and 20 (31.3%) newborns were admitted to the NICU. After excluding cases with poor image quality from the relevant regional analyses, segmentations were available for the cheeks in 64/64 cases (100%; AGA: 29/29, SGA: 35/35), trunk in 60/64 cases (93.7%; AGA: 29/29, SGA: 31/35), upper limbs in 59/64 cases (92.2%; AGA: 28/29, SGA: 31/35), and lower limbs in 60/64 cases (93.7%; AGA: 29/29, SGA: 32/35).

**Table 2:** Participants demographic data (*N*=64)

Characteristic	AGA (*n*=29)	SGA (*n*=35)
Maternal Age (years)	33.4 ± 4.2	34.3 ± 4.7
Maternal BMI at MRI (kg/m^2^)	26.3 ± 3.1	26.5 ± 4.4
Gestational diabetes	0, 0%	8, 22.8%
GA at MRI (weeks)	34 ± 1.8	33.9 ± 1.9
GA at birth (weeks)	37 ± 5	37 ± 1.9
Birth weight (g)	3269.6 ± 495.5	2110.8 ± 464.7
Fetal sex (female)	18, 62.1%	24, 68.6%
Delivery method		
Vaginal	18, 62.1%	17, 48.6%
Assisted	1, 3.4%	2, 5.7%
C-section	10, 34.5%	16, 45.7%
Apgar score		
1-min	9 (9–9)	9 (8–9)
5-min	10 (10–10)	10 (9–10)

Data are reported as mean ± standard deviation, median (interquartile range), or n, %

*AGA* appropriate for gestational age, *BMI* body mass index, *GA* gestational age, *SGA* small for gestational age

**Fig. 2:**
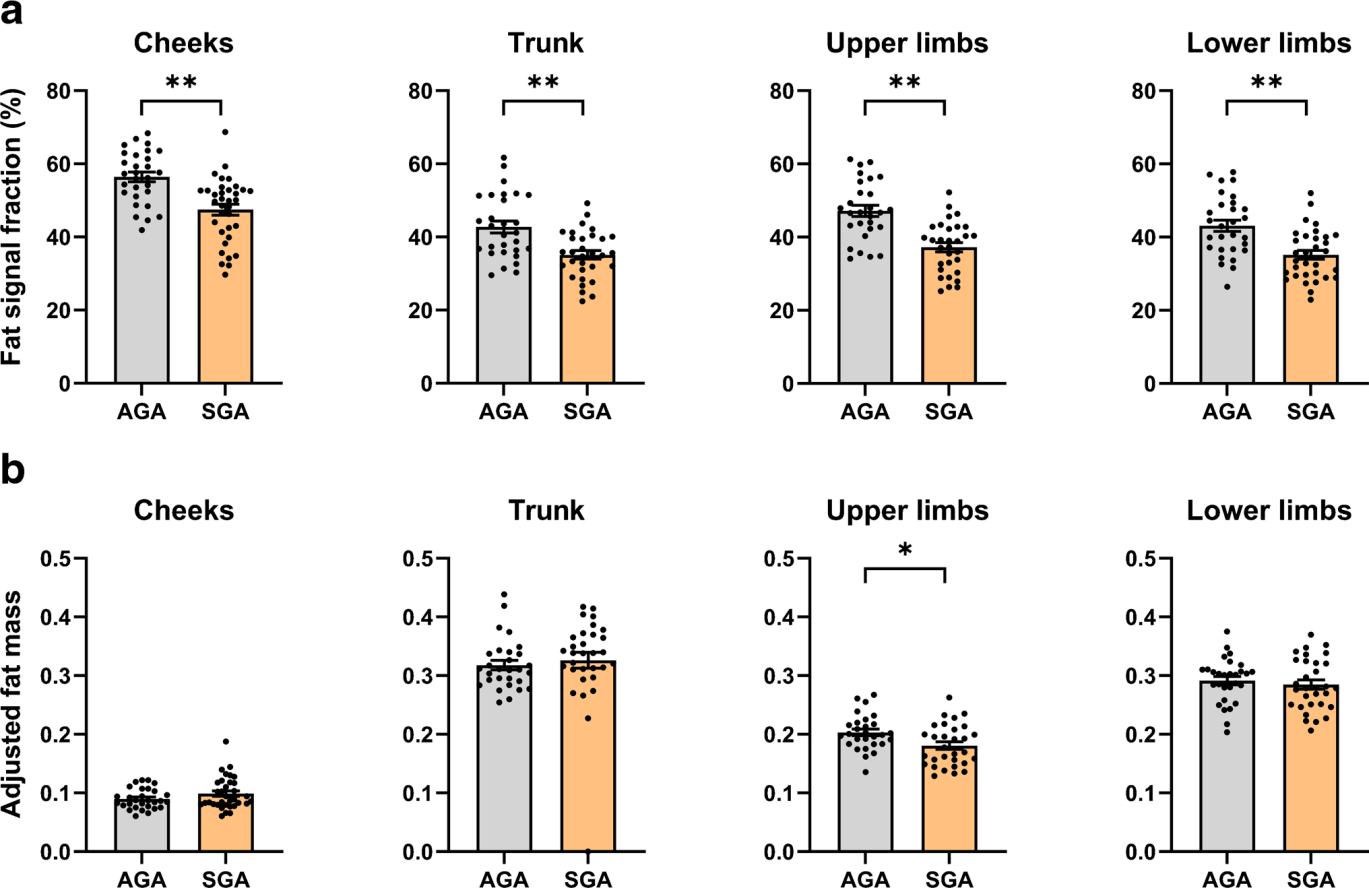
Differences in fat signal fraction (**a**) and adjusted fat mass (**b**) between participants with AGA and SGA-complicated pregnancies in fetal subcutaneous cheeks, trunk, upper and lower limbs. Bars represent mean values, whiskers represent standard error, and dots represent individual fetuses. Analysis was performed using mixed linear regression adjusted for fetal sex and gestational age. Benjamini–Hochberg correction was applied for multiple comparisons. *P<0.005, **P<0.001. *AGA* appropriate for gestational age, *SGA* small for gestational age

**Fig. 3:**
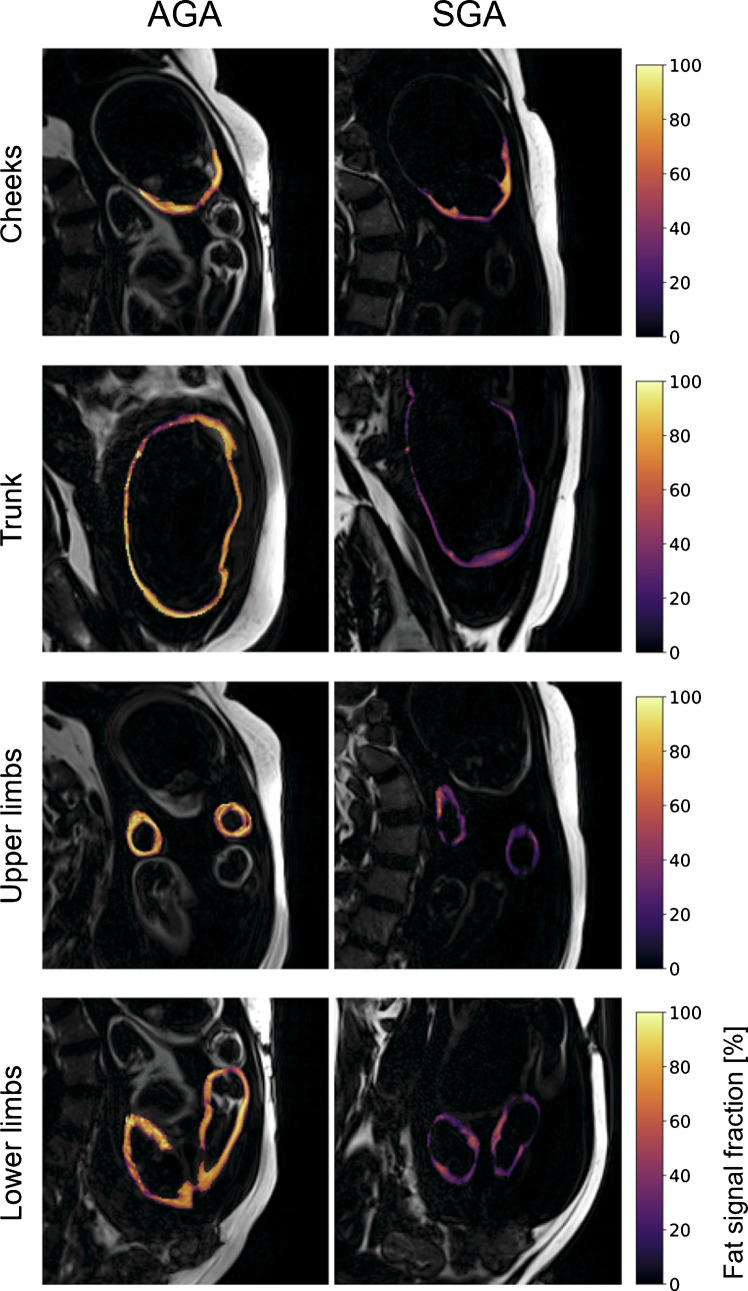
Illustration differences in adipose tissue deposition in two cases of AGA and SGA. MRI T_1_-weighted two-point Dixon fat-only image, the subcutaneous fat is segmented and color-coded according to the tissue’s fat signal fraction. The AGA case was a female fetus imaged at 33+5 weeks post conception and the SGA case was a female fetus imaged at 33+2 weeks post conception. The SGA case demonstrated reduced fat signal fraction and adipose tissue volume in all subregions. *AGA* appropriate for gestational age, *MRI* Magnetic resonance imaging, *SGA* small for gestational age

Figure 2 illustrates the differences between SGA and AGA in fat signal fraction and adjusted fat mass of the four body regions, and Supplementary Fig. 1 shows the relationship between GA and regional fat signal fraction and adjusted fat mass in both groups. Figure 3 demonstrates the differences in two representing cases of AGA and SGA. Table 3 lists the differences between SGA with early and late-onset disease and between cases with normal and abnormal CPR. Fat signal fraction was significantly lower in all subcutaneous body compartments among SGA fetuses compared with AGA controls (*P*<0.001). On average, fat signal fraction was 9.1% lower in the cheeks, 9.3% lower in the trunk and lower limbs, and 11.9% lower in the upper limbs among SGA compared with AGA. There were no significant differences in fat signal fraction between early- vs late-onset SGA (*P*=0.40) or between cases with normal vs abnormal CPR (*P*=0.54).

**Table 3:** Differences in fat signal fraction and adjusted fat mass between small-for-gestational-age cases with early (< 32 weeks) and late-onset (≥ 32 weeks) diagnosis and normal (≥ 5th centile) and abnormal (< 5th centile) CPR

Parameter	Region	Early (*n*=20)	Late (*n*=15)	Normal CPR (*n*=27)	Abnormal CPR (*n*=7)
Fat signal fraction	Cheeks	44.63 ± 1.85	46.35 ± 2.56	47.64 ± 1.46	43.34 ± 3.09
	Trunk	31.33 ± 1.9	34.63 ± 2.61	33.1 ± 1.47	32.86 ± 3.22
	Upper limbs	33.07 ± 1.93	36.99 ± 2.64	35.28 ± 1.46	34.79 ± 3.3
	Lower limbs	31.69 ± 1.9	34.03 ± 2.61	33.66 ± 1.46	32.05 ± 3.22
Adjusted fat mass	Cheeks	0.11 ± 0.001	0.1 ± 0.01	0.1 ± 0.01	0.11 ± 0.02
	Trunk	0.34 ± 0.01	0.36 ± 0.02	0.33 ± 0.01	0.37 ± 0.02
	Upper limbs	0.18 ± 0.01	0.2 ± 0.02	0.18 ± 0.01	0.2 ± 0.02
	Lower limbs	0.28 ± 0.01	0.29 ± 0.01	0.29 ± 0.01	0.28 ± 0.02

Results are presented as mean ± SE. Table 3 lists the differences in fat signal fraction and adjusted fat mass between SGA cases with early (< 32 weeks) and late-onset (≥ 32 weeks) diagnosis and between those with normal (≥ 5th centile) and abnormal (< 5th centile) CPR. Differences between groups was tested using linear mixed models while accounting to fetal sex and gestational age. There were no significant differences between the participants with early- and late SGA onset (*P*≥0.18) and between participants with normal and abnormal CPR (*P*≥0.052)

*CPR* cerebroplacental ratio, *SGA* small for gestational age

The adjusted fat mass of AGA fetuses was 1.11-fold higher in the upper limbs compared with SGA fetuses (*P*=0.043). There were no significant differences in the adjusted fat mass between SGA and AGA in the cheeks, trunk, and lower limbs (*P*>0.08). There were no significant differences in the adjusted fat mass between early- and late-onset SGA for all body compartments (*P*=0.18), and between SGA cases with normal and abnormal CPR (*P*=0.052).

Table 4 lists the results of the univariate logistic regression analyses assessing the associations of fat signal fraction and regional adjusted fat mass with composite adverse neonatal outcomes. There was a significant association between the fat signal fraction of the cheeks (*P*=0.02), trunk (*P*=0.026), UL (*P*=0.02), and LL (*P*=0.036) and perinatal morbidity. Conversely, none of the regional adjusted fat mass had a significant association with composite adverse neonatal outcomes (cheeks, *P*=0.084; trunk, *P*=0.57; UL, *P*=0.72; LL, *P*=0.62).

**Table 4:** Association between regional fat signal fraction and adjusted fat mass with composite adverse neonatal outcomes

Parameter	Region	OR (95% CI)	*P*	Adjusted *P*
Fat signal fraction	Cheeks	0.91 (0.85–0.97)	0.005	0.02
	Trunk	0.9 (0.83–0.98)	0.015	0.026
	Upper limbs	0.9 (0.83–0.97)	0.01	0.02
	Lower limbs	0.92 (0.85–0.99)	0.024	0.036
Adjusted fat mass	Cheeks	1.26 (0.99–1.6)	0.063	0.084
	Trunk	0.97 (0.89–1.06)	0.47	0.57
	Upper limbs	0.97 (0.83–1.14)	0.72	0.72
	Lower limbs	1.04 (0.91–1.19)	0.57	0.62

ORs and 95% confidence intervals (CI) refer to a 1% change in fat signal fraction or adjusted fat mass. Associations between regional fat signal fraction/adjusted fat mass and composite adverse neonatal outcomes were assessed with univariate logistic regression. P-values were corrected for multiple comparisons using the Benjamini–Hochberg procedure

*CI* confidence interval, *OR* odds ratio

## Discussion

In this study, we used fetal fat–water MRI scans to quantify regional adipose tissue in fetuses with SGA and compared them with AGA controls. We found that fat signal fraction was consistently reduced across all subcutaneous compartments in SGA fetuses compared with AGA, with the most pronounced differences in the upper limbs. We further demonstrated that the adjusted fat mass of the upper limbs was significantly reduced in SGA fetuses compared with AGA fetuses, but not in the other compartments. No significant differences were observed between early-onset and late-onset SGA, or between cases with normal and abnormal CPR.

Previous prenatal MRI studies have demonstrated progressive fat tissue growth during late gestation, both in volume and lipid fraction [7, 8]. Giza et al. further showed that fetal adipose tissue develops in a characteristic spatiotemporal pattern, beginning in the cheeks, followed by the proximal extremities and trunk, and ultimately the distal extremities and abdomen [8]. Notably, fat in the upper limbs accrues at the highest rate among all body regions[8].

Previous studies have shown that fetuses with fetal growth restriction or SGA exhibit a leaner body habitus, particularly those with fetal growth restriction [3]. Moreover, fetuses with reduced adiposity are more likely to require emergent delivery for non-reassuring fetal status and are at increased risk of neonatal morbidity [5, 6]. In our study, we observed significant regional differences in fat accretion between SGA and AGA fetuses. Notably, the upper limb adjusted fat mass was markedly reduced in SGA, reinforcing the notion that regions characterized by rapid third-trimester fat deposition are particularly susceptible to impaired growth. This observation is supported by prior ultrasound-based data demonstrating that fetal arm fat area is more strongly associated with newborn percentage body fat than thigh or abdominal fat measures, highlighting the upper limb as a potentially sensitive site for detecting altered neonatal adiposity and nutritional compromise [22]. Notably, the statistical significance of the upper limb adjusted fat mass difference was modest (*P*=0.043), and this finding should be interpreted with caution. However, it is biologically plausible, as prior work has shown that upper limb adipose tissue exhibits the most rapid fat accretion during the third trimester [8]. Regions with higher metabolic activity and growth velocity may be more sensitive to undernutrition, potentially explaining the preferential involvement of the upper limbs in SGA fetuses.

While adjusted fat mass demonstrated a selective regional difference, fat signal fraction was lower across all examined subcutaneous compartments, suggesting a more global reduction in adipose lipid content. The most pronounced reduction was again observed in the upper limbs, in keeping with the adjusted fat mass findings and supporting the concept that this rapidly accreting depot may be especially susceptible to growth impairment. Figure 3 presents representative single-slice images. The visual pattern is consistent with the quantitative analyses, which demonstrated lower fat signal fraction across all examined regions in SGA. While we hypothesized that earlier onset and abnormal CPR would be associated with more pronounced deficits, no notable differences in fat signal fraction or adjusted fat mass were observed between early- and late-onset SGA or between fetuses with normal versus abnormal CPR. This finding suggests that impaired adipose accretion may be a general hallmark of growth restriction, independent of diagnostic timing or circulatory redistribution. Nevertheless, the absence of subgroup differences may reflect limited sample size, heterogeneity in disease severity, or because regional adiposity metrics may not be sensitive enough to detect nuanced differences across these subclassifications.

In our cohort, regional fat signal fraction across all body regions was consistently associated with adverse neonatal outcomes, with ORs ranging from 0.90 to 0.92. Although this highlights fat signal fraction as a potentially sensitive marker of fetal nutritional status and a candidate biomarker for neonatal morbidity, no single body region appeared to provide greater predictive value than others. This aligns with prior work showing that whole-body fat signal fraction correlates with composite adverse neonatal outcomes [5]. In contrast, none of the regional adjusted fat mass measures were significantly associated with composite adverse neonatal outcomes. Previous studies similarly reported that the fetal fat-to-body volume ratio does not correlate with neonatal morbidity [5]. It remains unclear, however, whether this reflects a true lack of association between absolute adipose tissue volume and neonatal morbidity, or whether regional adjusted fat mass, normalized to total adipose volume, is less sensitive to perinatal risk than fat signal fraction.

The observation that fat signal fraction is uniformly reduced across all compartments in SGA fetuses underscores the systemic nature of nutrient deprivation rather than a region-specific pattern of fat signal depletion. Adipose tissue is highly responsive to caloric intake and substrate availability. Thus, impaired lipid accretion likely reflects reduced substrate delivery. From a physiologic perspective, the relatively greater reduction in the rapidly developing upper limb depot may indicate that accelerated growth regions are disproportionately affected when metabolic resources are limited. This pattern parallels observations in children [23] and adults [24] with low birthweight, who typically demonstrate preferential preservation of centrally located depots over appendicular fat stores. However, whether such postnatal patterns originate from differential prenatal adipose growth remains unknown and merits further exploration through longitudinal studies. Integrating these region-specific patterns with known metabolic adaptations of fetal growth restriction/SGA fetuses may provide further insights into how early-life nutritional insults shape neonatal and long-term metabolic trajectories.

Our findings also reinforce a previous study [5] that demonstrated the potential utility of adipose tissue fat fraction as a complementary imaging biomarker in the clinical evaluation of suspected growth restriction. Current tools, primarily ultrasound biometry and Doppler indices, capture fetal size and hemodynamic adaptation but do not directly assess the fetal metabolic reserve. Fat–water MRI, and fat fraction in particular, may offer a unique window into fetal energy stores, enabling more nuanced risk stratification beyond traditional metrics. A major barrier to broader clinical adoption is the need for image segmentation, which is traditionally labor- and time-intensive. However, the emergence of automated segmentation methods, including tools capable of generating real-time or near real-time measurements, may substantially enhance feasibility and support future integration into clinical workflows [25, 26].

Several limitations should be acknowledged. First, the study cohort of 64 participants is relatively small. The subgroup analyses according to SGA onset timing and CPR status included even smaller numbers, which may have limited statistical power to detect between-group differences. In addition, AGA participants were retrospectively identified from outpatient referrals. Although cases with genetic abnormalities, fetal infections, or abnormal fetal brain MRI were excluded to ensure a healthy control cohort, some individuals with AGA growth may still have been influenced by the clinical indication prompting the MRI referral. Furthermore, gestational diabetes was present in some SGA pregnancies and may have influenced fetal adipose tissue measurements, representing a potential confounder. Fetal body composition is also known to vary across populations [27], which may affect the generalizability of our findings. Although regional segmentation failures due to image quality were infrequent, they occurred more often in the SGA group; therefore, a small degree of selection bias in the affected regional analyses cannot be excluded. In addition, although the deep-learning automatic segmentations substantially reduced segmentation burden, manual correction and quality control were still required, introducing potential observer-dependent variability. We used a two-point Dixon acquisition sequence with a relatively large flip angle of nine degrees, which is susceptible to magnetic field inhomogeneity as well as T_1_ and T_2_^*^ bias. As a result, the fat signal fraction values reported here should be interpreted as estimations of tissue lipid content rather than precise quantitative measurements. Lastly, despite the relatively small voxel size (1.25–1.4 × 1.25–1.4 × 2 mm), partial volume effects at adipose tissue boundaries may have influenced the measured fat fraction and fat mass and should be considered a technical limitation of this study.

In conclusion, fetuses that are SGA exhibit significantly reduced subcutaneous adipose tissue lipid fraction across all compartments compared with AGA controls, with a more pronounced fat mass reduction in the upper limbs. These findings emphasize the systemic nature of impaired fetal fat accretion in fetal growth restriction/SGA and suggest relative preservation of central fat depots compared with appendicular, rapidly developing regions. Importantly, fat signal fraction provides prognostic information beyond fetal size, reflecting tissue-level metabolic alterations. These results highlight the value of MRI-derived adiposity metrics in revealing subtle metabolic consequences of impaired fetal growth that are not captured by conventional imaging. By characterizing how SGA is associated with disrupted regional fat accretion, this work provides a foundation for integrating metabolic markers into future fetal assessment frameworks and may help refine our understanding of how early nutritional deficits shape neonatal risk.

## Supplementary Information

Below is the link to the electronic supplementary material.

ESM 1 (DOCX 114 KB)

## Data Availability

The data underlying this article cannot be shared publicly due to the privacy of individuals who participated in the study. The data will be shared on a reasonable request to the corresponding author.
