# Supplementary material for: Regional differences in fetal fat accretion in small-for-gestational-age fetuses assessed by quantitative magnetic resonance imaging
Source: Pediatr Radiol. 2026 Jul 1;56(8):1803–13. doi: 10.1007/s00247-026-06702-2 (PMC13407942; doi:10.1007/s00247-026-06702-2)
Supplement: Supplementary file 1 — (DOCX 114 KB) [file 247_2026_6702_MOESM1_ESM.docx]

**Supplementary material for “Regional differences in fetal fat accretion in small-for-gestational-age fetuses assessed by quantitative magnetic resonance imaging”**


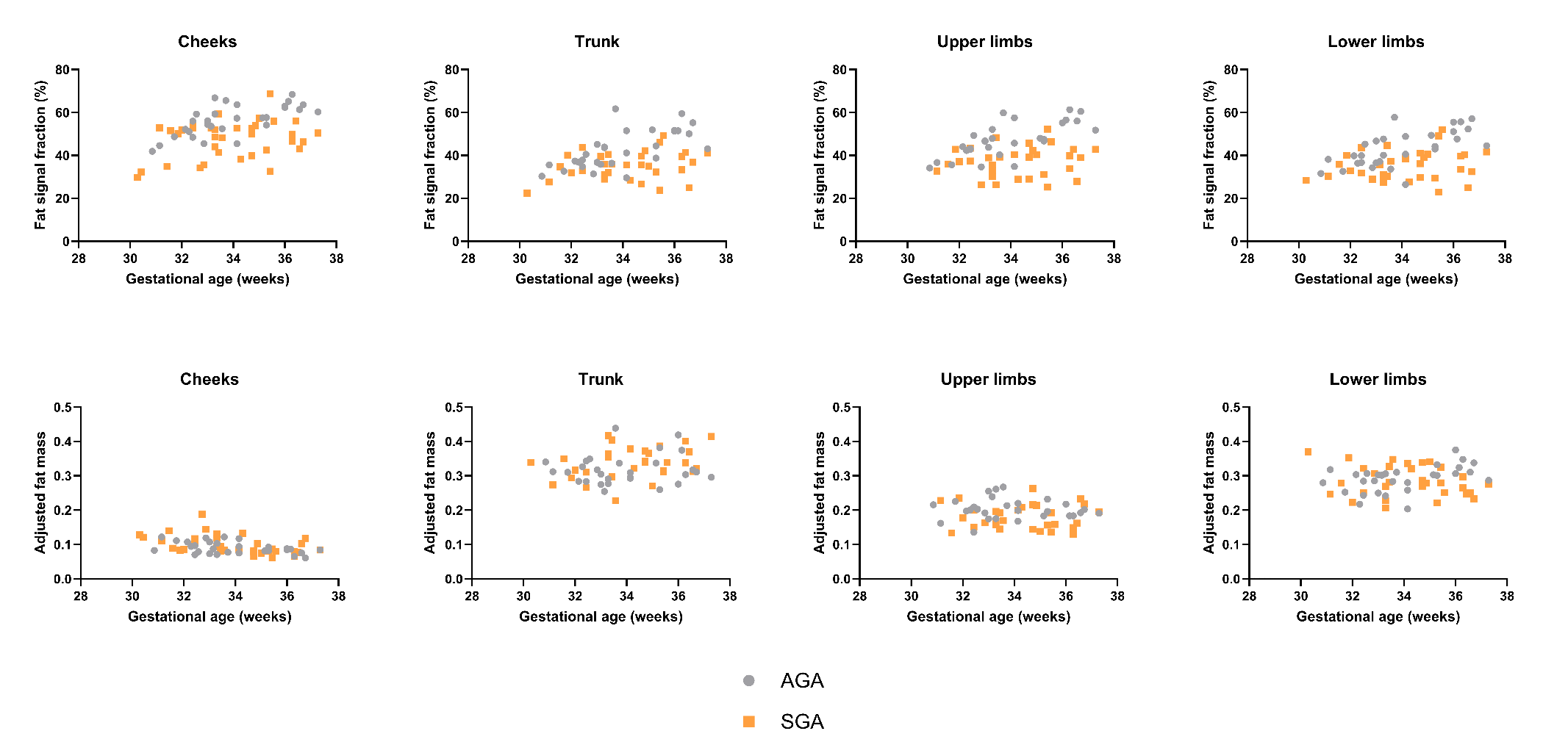


Supplementary Figure 1. Relationship between gestational age and regional fetal subcutaneous adiposity in AGA and SGA pregnancies. Scatter plots demonstrate fat signal fraction and adjusted fat mass across gestational age for the cheeks, trunk, upper limbs, and lower limbs. *AGA* appropriate for gestational age, *SGA* small for gestational age
